# Artificial Intelligence in microbiomes analysis: A review of applications in dermatology

**DOI:** 10.3389/fmicb.2023.1112010

**Published:** 2023-02-01

**Authors:** Te Sun, Xueli Niu, Qing He, Fujun Chen, Rui-Qun Qi

**Affiliations:** ^1^Department of Dermatology, The First Hospital of China Medical University, Shenyang, China; ^2^Key Laboratory of Immunodermatology, Ministry of Education and NHC, National Joint Engineering Research Center for Theranostics of Immunological Skin Diseases, Shenyang, China; ^3^Liaoning Center for Drug Evaluation and Inspection, Shenyang, China

**Keywords:** Artificial Intelligence, microbiome, machine learning, convolutional neural networks, microbial sequencing, gut-skin axis

## Abstract

Microorganisms are closely related to skin diseases, and microbiological imbalances or invasions of exogenous pathogens can be a source of various skin diseases. The development and prognosis of such skin diseases are also closely related to the type and composition ratio of microorganisms present. Therefore, through detection of the characteristics and changes in microorganisms, the possibility for diagnosis and prediction of skin diseases can be markedly improved. The abundance of microorganisms and an understanding of the vast amount of biological information associated with these microorganisms has been a formidable task. However, with advances in large-scale sequencing, artificial intelligence (AI)-related machine learning can serve as a means to analyze large-scales of data related to microorganisms along with determinations regarding the type and status of diseases. In this review, we describe some uses of this exciting, new emerging field. In specific, we described the recognition of fungi with convolutional neural networks (CNN), the combined application of microbial genome sequencing and machine learning and applications of AI in the diagnosis of skin diseases as related to the gut-skin axis.

## Introduction

A tremendous array of microorganisms widely exist in nature and the human body. In healthy humans various types of microorganisms and dominant microbiota are present within different parts of the body and a stable number and ratio of microorganisms are maintained through competition or synergy (Dominguez-Bello et al., 2019). With changes in the ratio between the dominant microbiota and individual microbiota, a microecological imbalance occurs, which can lead to specific skin diseases and, the characteristics of these changes are related to the progression of the disease. Due to the large number and variety of microorganisms, analyzes using previous techniques have been incapable of handling these data (Goodswen et al., 2021). However, with the development of AI and machine learning related technologies, information based on microbial image recognition or genomics data can be applied in many fields, including the identification of specific conditions for application in forensic science and clinical disease diagnosis. In this review, we provide a detailed introduction to the application of AI as based on microbial information for use in diagnosing skin diseases and predicting disease progression of these conditions.

## Recognition of fungi with convolutional neural networks

Convolutional Neural Network (CNN) is a special type of machine learning that can assimilate both isolated topographies as well as entire images and classify these images according to their unique features (Dildar et al., 2021). Dermatologists can also apply this method for disease diagnosis. For example, an image is annotated according to the corresponding medical records and pathological results and, after generating standardized data, these data can then be analyzed using CNN to distinguish and thus diagnose skin lesion images from that of normal skin images (Haenssle et al., 2018; Liopyris et al., 2022). This method is often used in the diagnosis of fungal infections, such as onychomycosis, as this condition represents the most common nail disease infected with fungi. The traditional clinical diagnosis for this condition is based on direct microscopy with potassium hydroxide (KOH), a periodic acid schiff stain (PAS) and/or a fungal culture. However, as colony formation requires an extended period of time and is susceptible to antifungal drugs, this diagnostic approach can be problematic (Gupta et al., 2020). The diagnosis of this condition, which involves the observation of a specific type of fungal morphology, is particularly suitable for that of CNN (Hogarty et al., 2020; Zhang et al., 2021).

The differences in diagnosing onychomycosis using CNN vs. manual microscopic examinations have been compared and analyzed. Results from one report found that the accuracy of CNN diagnosis was 10% greater than that of traditional diagnostic methods (Yilmaz et al., 2022). In that study, 60 nail samples from patients with onychomycosis and 297 nail samples from healthy controls were treated with KOH. Two different CNN diagnostic performance models (VGG16 and InceptionV3) were developed. These two models have different algorithms, but both accomplish the purpose of diagnosis by extracting fungal-specific structures. As compared with that of the traditional clinical method, these two CNN models not only demonstrated a higher degree of accuracy, but also showed a better sensitivity (75.04% and 74.93% vs. 74.81%) and specificity (92.67% and 93.78% vs. 74.25%). Similar findings were reported in another study with the specificity of diagnosis using VGG16 being 72.7% vs. 49.3% with the traditional clinical method, however, the sensitivity of CNN diagnosis was slightly lower at 70.2% vs. 73%, as determined in a group of 90 patients (Kim et al., 2020). In addition, CNN showed a greater degree of specificity in the diagnosis of onychomycosis as compared with conventional diagnostic methods using the periodic acid-Schiff reaction (PAS stain). As reported in a study with 199 cases, CNN showed an increased level of specificity (98% vs. 90.35%) and area under the receiver operating characteristic curve (AUC – 0.960 vs. 0.932) as compared to that obtained with three dermatopathologists (Decroos et al., 2021). Moreover, CNN can also be used as a primary screening tool to assist manual microscopic examinations to greatly improve diagnostic accuracy. As the specificity and sensitivity of machine learning can be adjusted by changing the intersection over union (IOU) parameter, the specificity of machine learning can be increased by increasing IOU to ensure a higher true positive rate. Subsequently, the clinician can re-screen samples diagnosed as negative in hyphae with use of CNN to reduce the false negative rates. Such an approach not only improves detection efficiency and reduces expenses, but also increases diagnostic accuracy (Koo et al., 2021).

In addition to nails, fungal infections within other regions, such as the skin and hair, can also be diagnosed using CNN. However, due to the expansive areas involving skin and hair, the lesions are not concentrated. As a result, the low fungal content in an individual lesion hinders the recognition of mycelial characteristics by CNN, which can then decrease the diagnostic accuracy in these regions (Gao et al., 2021). One approach to alleviate the deficiency of CNN to extract effective information from small-scale data sets, is to combine the CNN model with the attention mechanism (AM) to build an IL-MCAM framework. IL-MCAM is based on attention mechanisms and interactive learning and can be applied to add misclassified images to the training sets using an interactive approach after the images have been classified with CNN to improve the classification ability of the CNN model. Although, to our knowledge, no reports are available using IL-MCAM to diagnose fungal skin diseases, a 99.77% correct diagnosis rate for colorectal cancer has been reported with this model (Chen et al., 2022a).

## Combined application of microbial genome sequencing and machine learning

Machine learning can provide the means for identifying patterns in the sequencing data of a pathogen to generate a system for subdividing that pathogen. In this way, it can be used to determine which branch of the pathogen is infected to provide a basis for administration of the most appropriate medication. This method can be applied for the diagnosis of syphilis, as the internal structure of treponema is like that of bacteria with five genera, among which treponema is the pathogenic bacteria resulting in syphilis. In contrast, the clinical diagnosis of syphilis requires serological tests including TPPA and TPHA, which lack the ability to determine which branch of the pathogen is infecting the patient, thus precluding decisions regarding the most effective medication (Forrestel et al., 2020). One example of this approach has been applied to enable an advanced determination as to whether a patient was infected with drug-resistant spirochetes. Investigators collected and sequenced treponema pallidum from syphilis-infected patients in 8 countries and 6 continents and classified the spirochete branches using machine learning – maximum likelihood phylogeny method (ss14 and Nichols). It was found that the clades recognized by this model as Nichols C and Nichols B were consistent with resistance to azithromycin (Lieberman et al., 2021). This observation not only helped in clinically diagnosing the type of syphilis, as achieved using PC, but also provided a guide with regard to the initial administrations of medications to avoid use of ineffective antibiotics for patients infected with drug-resistant strains. While promising, these findings were based on a small sample size which lacked South Asian and South American populations, which raises an issue regarding the reliability and validity of these experimental results.

Machine learning can process 16S sequencing results to obtain information on differences in microbial species and composition ratios between patients and healthy individuals. Compared with traditional diagnostic methods, machine learning has the capacity to obtain additional information regarding body skin status and pathogen type. The 16S sequencing approach is a commonly used sequencing method to reveal species composition and evolution, mainly *via* its ability to detect partial fragments of microbial ribosomal DNA. This fragment includes 9 variable and 10 conserved regions, with the former determining relationships between species and the latter providing an understanding of differences between species. Diversity information of microorganisms in a sample can be obtained by amplifying and sequencing partial regions of the rRNA DNA sequence in the extracted sample (Abellan-Schneyder et al., 2021). As an example, males with an HPV infection may present with insidious symptoms, but their penile microbiota will change, with this change placing their sexual partners at risk for HPV infection (Onywera et al., 2020b). The V3-V4 hypervariable regions of the 16S rRNA gene from the penile skin microbiota of 238 South African males were analyzed, and 6 distinct community state types (CSTs) were identified. With use of the machine learning – linear discriminant analysis effect size algorithm, differences in the abundance of microbial populations were observed as a function of different HPV infection subtypes. High-risk (HR)-HPV males had a significantly greater relative abundance of prevotella, dialister, peptoniphilus, and unclassified clostridiales and CST types 2–6, as compared with those not infected with HR-HPV. Males with a CST type dominated by corynebacterium were less likely to be infected with HR-HPV, but the opposite was true for women (Onywera et al., 2020a). While all HPVs are contagious, different types of HPV infection can lead to different diseases including genital warts, flat warts and even genital cancer. Due to its mode of transmission (contact transmission) and the potential for latent clinical symptoms following infection, HPV infections can readily affect the health of sexual partners, if no treatments and/or protection are undertaken. Therefore, 16S sequencing and machine learning represent important tools which can be used to predict, not only the HPV type, but even the type in their partner, which enables the possibility for an early detection and treatment.

In addition to being used in the diagnosis of HPV infection, 16S sequencing has also been used in evaluating skin status and generating probabilities for the prediction of skin diseases. In one study, 1,200 microbial samples were obtained from the legs of Canadian women aged 21–65 and subjected to 16S rRNA sequencing. Combined with skin hydration status (including PH value and conductance capacitance), three machine learning methods – random forest (RF), XGBoost, and LightGBM were used to analyze these samples. In addition, samples from the legs of 278 British women were also obtained for analysis using machine learning. The results from this study revealed that skin moisture levels were higher and a better skin condition was observed as a function of increased levels of lactobacilli. With an abundance of Bergeyella, the skin was dehydrated and the probability for dermatitis was relatively high (Carrieri et al., 2021).

Metagenomics, which differs from that of 16S sequencing, directly extracts DNA from all microorganisms of environmental samples, with the detection object including all microbial genomes, due to its more prolific genome database (Gu et al., 2019). The diagnosis of acne can be performed by analyzing the metagenomic sequencing data of acne using machine learning methods. Acne, which is associated with adipogenic fibroblasts, genetic factors and skin and intestinal microbiota, is one of the most common skin diseases worldwide (Mitchell et al., 2022; Sánchez-Pellicer et al., 2022). As it remains unclear whether changes in skin microbiota play an indicative role in acne, diseased skin (DS) and healthy skin (HS) samples from 35 acne patients and 35 normal control (NC) skin samples were collected for analysis. Through metagenomics analysis, 2,520 sequence data points from each volunteer were selected. Using machine learning – principal component analysis (PCA) and kernel principal component analysis (KPCA) methods, the corresponding lipids that largely contributed to the status of each type of skin were identified. Using a multiset canonical correlation analysis (MCCA) method, lipids which can effectively differentiate among the three different skin states were revealed, with the results that lipid No. 1240 can distinguish a DS sample set, lipids No. 608 and 2334 can distinguish a HS sample set and a decrease in lipids No. 95, 1069, and 1108 indicates an improvement in the disease. Accordingly, the results of this study have significant implications with regard to the diagnosis of acne (Wang et al., 2021).

## AI and skin diseases as related to the gut-skin axis

Gut microbiota play an important role in maintaining human health. The host and microbiota maintain a state of homeostasis within the body through subtle interactions, with disruptions in this balance affecting the entire organism, even within organs far removed from the gut, such as the integumentary system (De Pessemier et al., 2021). In fact, increasing evidence has accrued which indicates that many skin diseases are accompanied by alterations in the gut microbiome (e.g., atopic dermatitis, psoriasis, vitiligo, and acne vulgaris; Szántó et al., 2019). Such findings have led to development of the gut-skin axis concept. That is, when the relationship between gut microbes and the immune system is compromised, subsequent effects on the skin can be triggered and even develop into skin diseases. Therefore, skin diseases may be diagnosed through the detection of gut microbes (Mahmud et al., 2022).

Results from previous studies have shown that microbes on the skin surface are highly related to the occurrence and development of vitiligo, and the progression of this disease can be estimated by observing changes in skin microbes. For example, increased levels of streptomycin and streptococci are observed in active vs. stable vitiligo as detected by the Novaseq sequencer; and differences in Beta diversity (Non-Metric Multi-Dimensional Scaling) are present between patients with active vs. stable vitiligo (Lu et al., 2021). While the composition of gut microbiomes remains stable from infancy, skin surface microbes are susceptible to environmental influences. Only in the presence of vitiligo does the proportion of microorganisms in gut microbes change as a function of disease progression. When gut microbes of 30 patients with vitiligo were compared with that of 30 matched healthy controls, results from the 16S rRNA sequencing assay revealed that the Shannon and Simpson index was higher and the ratio of Bacteroides/Firmicutes decreased in vitiligo patients (Ni et al., 2020). The alpha-diversity (a measure of richness and uniformity) in patients experiencing vitiligo for >5 years was greater than that of patients experiencing a shorter interval of illness. Finally, when combining machine learning with assay results indicating the presence of Corynebacterium 1 and Psychrobacter, a diagnosis of vitiligo with an accuracy rate of 0.929 was obtained. These data suggest that gut microbiota can not only be used to distinguish vitiligo in patients vs. healthy individuals, but can also provide a determination for the duration of this disease.

Atopic dermatitis (AD) is a chronic inflammatory disease that may result from a complex interaction among genetic predisposition, immune dysfunction, environmental allergens and skin barrier abnormalities. Interestingly, results from previous studies have suggested that AD patients show abnormal gut microbiomes prior to the onset of this disease. Infants (2 months of age) whose fecal calprotectin was greater than normal showed an increased risk of developing AD at 6 years of age (Lunjani et al., 2018). In addition, these children had increased *E. coli*, fewer bifidobacteria, bacteroides and lower levels of alpha-diversity (Arboleya et al., 2016). Such findings indicate the importance of intestinal microbial changes in the diagnosis of AD as can be determined using machine learning (Jiang et al., 2022). In that study, data from intestinal epithelial cell transcriptomes and flora were collected from 88 AD patients and 73 healthy controls (the average age of the healthy group was 3 months younger than that of the AD group) and the supervised machine learning pipeline—Logistic Regression (LR), Support Vector Machine (SVM), and Random Forest Classifier (RFC) were constructed as based on 44,608 gene expression probes and 366 species of microorganisms in transcriptome and microbial databases. Fifty microbial characteristic maps as related to AD were screened, including akkermanisia, verrucomicrobia, propionibacterium, and those with the highest F1 scores (high precision 0.70 and recall 0.88), could then be used as AD predictors (Jiang et al., 2022). Finally, results from a literature review have verified that these microbial characteristics are highly correlated with AD, and therefore cannot only be used to predict AD but even distinguish among disease subtypes.

## Discussion

The human microbiome is closely related to skin diseases. Accordingly, an understanding of the microbial community composition, structure, function and its changes within the skin can serve as critical indices for the diagnosis of skin diseases. With this method, different machine learning models can be used to analyze changes in the abundance, type, and composition ratio of microorganisms in different aspects of the disease state versus that of healthy people, which can then enhance the accuracy of diagnosing skin diseases. Moreover, as compared with that of traditional methods, this procedure can also serve to predict the occurrence and progression of diseases.

As this new technology currently resides in developmental stages, many limitations remain. For example, sequencing data have low rates of representation and insignificant features as well as easy under-segmentation caused by the image characteristic which can result in a reduction in the accuracy of conclusions derived with this technique (Zhang et al., 2022). In addition, it is difficult for CNN to identify spores and hyphae with varying degrees of linear curvature and the relatively low resolution of pathological images can make it difficult for AI to distinguish between serum particles and fungal elements in the images. However, this reduction in accuracy resulting from low-resolution images can be resolved by altering the method used for image generation, such as using whole slide imaging (WSI) or through application of visual transformer models to obtain more stable results (Chen et al., 2022b; Li et al., 2022). The development of 269 AI has accelerated the progress of research in the study of microorganisms. In addition to the capacity for disease diagnosis and prediction as described in this report, AI’s automatic identification of microbial genomes can also be applied for forensic identification of deceased individuals and thus offers the potential to assist forensic investigators in resolving medical disputes. While combining AI with microbes remains a challenge, this technology holds great promise for applications in the fields of medicine and forensics.

## Author contributions

TS: searched and analyzed the published literature and drafted the manuscript. XN and QH: searched and analyzed the published literature. R-QQ and FC: reviewed and edited the manuscript. All authors contributed to the article and approved the submitted version.

## Funding

This study was funded and supported by National Natural Science Foundation of China (82173401 and U1908206).

## Conflict of interest

The authors declare that the research was conducted in the absence of any commercial or financial relationships that could be construed as a potential conflict of interest.

## Publisher’s note

All claims expressed in this article are solely those of the authors and do not necessarily represent those of their affiliated organizations, or those of the publisher, the editors and the reviewers. Any product that may be evaluated in this article, or claim that may be made by its manufacturer, is not guaranteed or endorsed by the publisher.
